# Trained immunity is induced in humans after immunization with an adenoviral vector COVID-19 vaccine

**DOI:** 10.1172/JCI162581

**Published:** 2023-01-17

**Authors:** Dearbhla M. Murphy, Donal J. Cox, Sarah A. Connolly, Eamon P. Breen, Aenea A.I. Brugman, James J. Phelan, Joseph Keane, Sharee A. Basdeo

**Affiliations:** 1 Human and Translational Immunology Group, School of Medicine,; 2 Tuberculosis Immunology Group, Department of Clinical Medicine, and; 3 Core Facilities, Trinity Translational Medicine Institute, St. James’s Hospital, Trinity College Dublin, The University of Dublin, Dublin, Ireland.

**Keywords:** COVID-19, Vaccines, Cytokines, Glucose metabolism, Monocytes

## Abstract

**Background:**

Heterologous effects of vaccines are mediated by “trained immunity,” whereby myeloid cells are metabolically and epigenetically reprogrammed, resulting in heightened responses to subsequent insults. Adenovirus vaccine vector has been reported to induce trained immunity in mice. Therefore, we sought to determine whether the ChAdOx1 nCoV-19 vaccine (AZD1222), which uses an adenoviral vector, could induce trained immunity in vivo in humans.

**Methods:**

Ten healthy volunteers donated blood on the day before receiving the ChAdOx1 nCoV-19 vaccine and on days 14, 56, and 83 after vaccination. Monocytes were purified from PBMCs, cell phenotype was determined by flow cytometry, expression of metabolic enzymes was quantified by RT-qPCR, and production of cytokines and chemokines in response to stimulation ex vivo was analyzed by multiplex ELISA.

**Results:**

Monocyte frequency and count were increased in peripheral blood up to 3 months after vaccination compared with their own prevaccine controls. Expression of HLA-DR, CD40, and CD80 was enhanced on monocytes for up to 3 months following vaccination. Moreover, monocytes had increased expression of glycolysis-associated enzymes 2 months after vaccination. Upon stimulation ex vivo with unrelated antigens, monocytes produced increased IL-1β, IL-6, IL-10, CXCL1, and MIP-1α and decreased TNF, compared with prevaccine controls. Resting monocytes produced more IFN-γ, IL-18, and MCP-1 up to 3 months after vaccination compared with prevaccine controls.

**Conclusion:**

These data provide evidence for the induction of trained immunity following a single dose of the ChAdOx1 nCoV-19 vaccine.

**Funding:**

This work was funded by the Health Research Board (EIA-2019-010) and Science Foundation Ireland Strategic Partnership Programme (proposal ID 20/SPP/3685).

## Graphical abstract

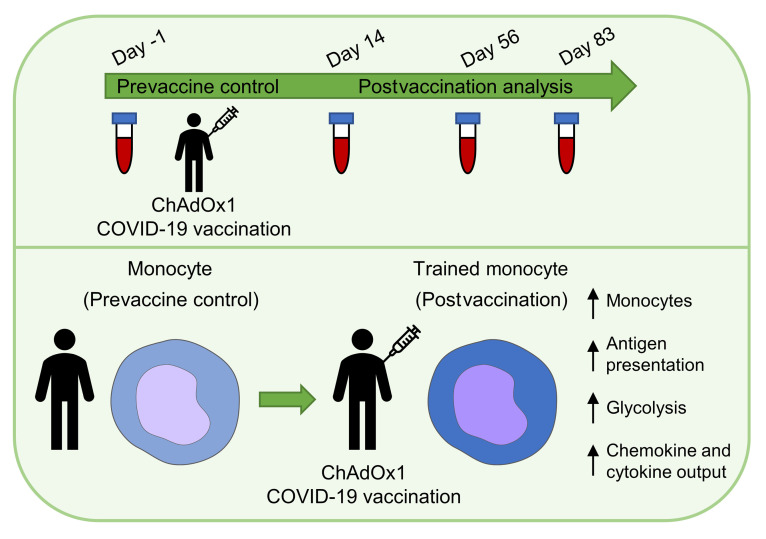

## Introduction

The vaccine effort against SARS-CoV-2 was unprecedented and highly successful, largely due to the vast body of preexisting research and development in the field. Vaccine design focused on developing adaptive immune responses against SARS-CoV-2 through the production of neutralizing antibodies from B cells and through the generation of memory T cells, which elicit protection against SARS-CoV-2 severity and mortality. The innate immune response is critical to the orchestration of protective adaptive immune responses after vaccination. A growing body of evidence now indicates that the mammalian innate immune response has a capacity for a type of memory termed “trained immunity” (1). The role that trained immunity plays in vaccine efficacy remains unknown. However, trained immunity mediates the nonspecific protective effects of live attenuated vaccines, such as the BCG vaccine against tuberculosis (2–5), which is known to reduce all-cause mortality in infants (6–9). This occurs because the vaccine induces epigenetic and metabolic rewiring of monocytes, which leaves them primed to respond in a heightened manner when they are subsequently stimulated (2, 3, 10). Importantly, this effect outlives the short time frame of immune activation subsequent to vaccination and has been attributed to changes in the bone marrow hematopoietic stem cell niche, which result in enhanced myelopoiesis and the egress of myeloid cells that are epigenetically and metabolically reprogrammed owing to trained immunity (5, 11). Comparisons among randomized clinical trials of COVID-19 vaccines suggest that adenovirus vector–based vaccines may have nonspecific protective effects resulting in significantly reduced all-cause mortality and non-COVID, nonaccident mortality compared with mRNA-based vaccines (12).

Notably, an intranasal adenoviral vaccine vector has been shown to induce trained immunity in the airways of mice and reduced disease burden when the mice were subsequently infected with *S*. *pneumoniae* or *M*. *tuberculosis* (13, 14). Furthermore, preliminary studies showed that vaccination of humans with an aerosolized adenovirus-vectored tuberculosis vaccine induced persisting transcriptional changes in alveolar macrophages (15). We sought to determine if the ChAdOx1 nCoV-19 vaccine (produced by AstraZeneca), which uses a replication-deficient simian adenovirus vector could induce trained immunity in vivo in humans. We drew venous blood from healthy adults the day before vaccination and 14, 56, and 83 days after vaccination with a single dose of the ChAdOx1 nCoV-19 vaccine. Monocyte phenotype and function was assessed at these time points after vaccination and compared with their own prevaccine baseline control sample.

Our results indicate that the ChAdOx1 nCoV-19 vaccine may enhance myelopoiesis up to 3 months after vaccination. Our data indicate that the vaccine induced metabolic reprogramming in monocytes, which was sustained at 2 months after vaccination. Moreover, monocytes exhibited enhanced antigen presentation functions and had increased capacity to produce key cytokines and chemokines in response to subsequent unrelated stimuli. Taken together, these data indicate that the ChAdOx1 nCoV-19 vaccine can induce prolonged innate immune activation, with many features of trained immunity.

## Results

**Figure 1:**
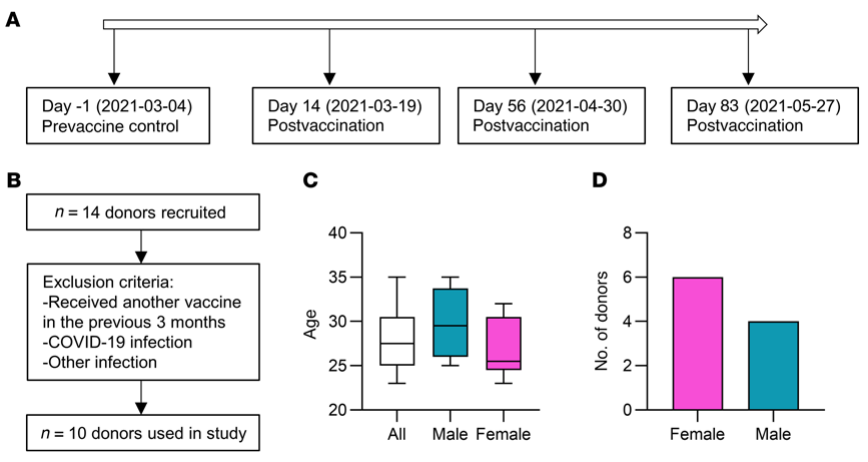
Schematic representing the study design and donor information. (**A**) Timeline used for sample acquisition. (**B**) Flow chart of donors recruited, the exclusion criteria, and the donors who were evaluable. (**C**) Graph showing the age, with mean and SD, of the total donor cohort (white), male donors (blue), and female donors (pink). (**D**) Number of donors recruited to the study stratified by sex.

Blood from healthy donors who received the ChAdOx1 nCoV-19 vaccine was collected the day before (day –1) and 14, 56, and 83 days after vaccination (Figure 1A). Donors were excluded from participating at any time point if they had received another vaccine in the last 3 months or if they knowingly got infected with COVID-19 or another infectious agent (Figure 1B). The age range of the participants was 23–35 years old (Figure 1C) and included 6 female and 4 male donors (Figure 1D). PBMCs were isolated and monocytes were enriched using a hyperosmotic Percoll gradient. Cells were examined by flow cytometry and RT-qPCR. Monocytes were stimulated ex vivo to assess their ability to respond to unrelated stimuli before and after vaccination. Concentrations of soluble inflammatory mediators present in the supernatants 24 hours after stimulation were determined by multiplex ELISA.

### CD14^^+^^ monocytes were increased up to 3 months after vaccination with ChAdOx1 nCoV-19, indicative of enhanced myelopoiesis.

**Figure 2:**
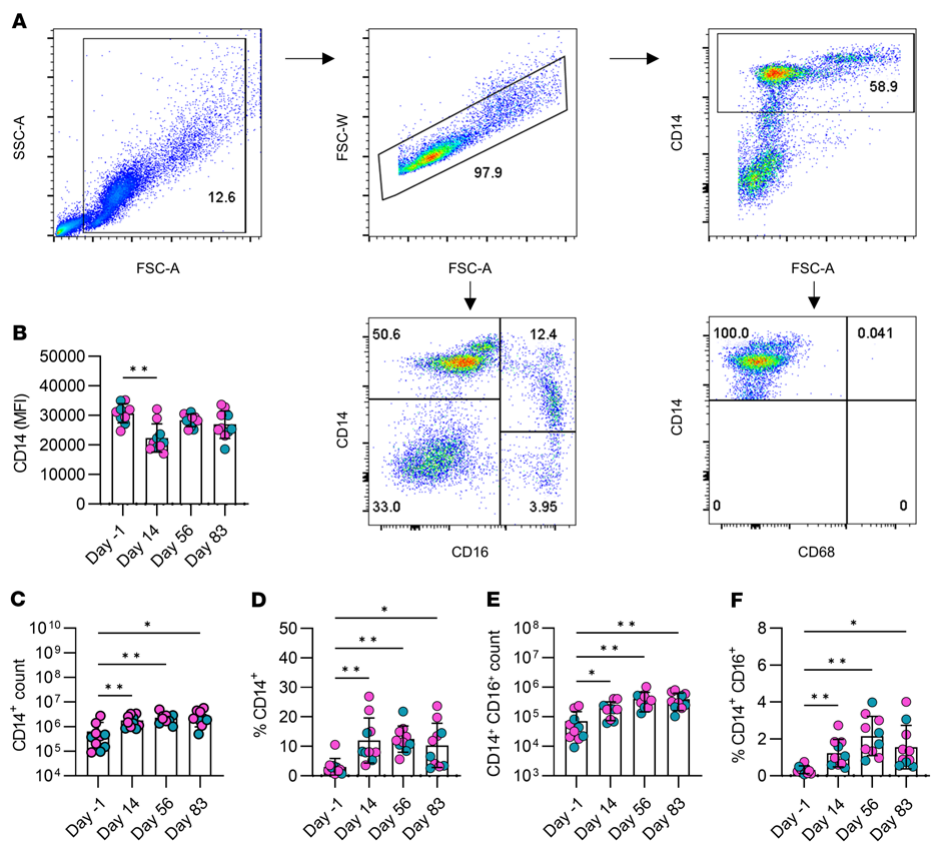
Long-term effects of vaccination on monocyte subsets in the blood. Monocytes were isolated from PBMCs from healthy donors on day –1 (prevaccine), day 14, day 56, and day 83 after vaccination (and prior to the second dose) using a hyperosmotic Percoll gradient. (**A**) Cells were Fc blocked and stained with fluorochrome-conjugated antibodies specific for CD14, CD68, and CD16. Total monocytes were identified as CD14^+^CD68^–^, and CD14^+^CD16^+^ monocytes were also examined. Numbers in the dot plots indicate the frequencies of cells (%) present inside the gate or quadrant. (**B**) The median fluorescent intensity of CD14 in the total ex vivo CD14^+^ population was assessed over time. (**C**) The absolute number of CD14^+^ cells was calculated by multiplying the total cell yield from the hyperosmotic Percoll enrichment by the percentage of CD14^+^ cells. (**D**) Monocyte frequency was calculated by dividing the total number of CD14^+^ cells by the total number of PBMCs. (**E**) The absolute number of CD14^+^CD16^+^ cells was calculated by multiplying the total cell yield from the hyperosmotic Percoll enrichment by the percentage of CD14^+^ CD16^+^ cells. (**F**) CD14^+^CD16^+^ monocyte frequency was calculated by dividing the total number of CD14^+^CD16^+^ cells by the total number of PBMCs. Each dot represents an individual donor (*n* = 10), with blue dots denoting male donors and pink dots denoting female donors. Data are graphed as the mean value ± SD. Statistically significant differences between the groups were determined by repeated measures 1-way ANOVA using Dunnett’s multiple comparisons test; *P < 0.05, **P < 0.01.

Increased myelopoiesis and modulation of myeloid progenitors is an essential mechanism underpinning trained immunity (5, 11, 16, 17) and mechanistically accounts for the longevity of the effects, because training outlasts the short life span of the original activated monocytes. Reprogramming of hematopoietic stem cells in the bone marrow results in monocytes with enhanced function against related and unrelated infections (5, 11, 16–18). To assess the effect of ChAdOx1 nCoV-19 vaccination on myelopoiesis, the absolute number and relative frequency of monocytes in the peripheral blood was assessed. Cells were Fc blocked and stained for fluorochrome-conjugated antibodies specific for CD14, CD16, and CD68 prior to acquisition by flow cytometry. Monocytes were gated as shown in the gating strategy (Figure 2A).

We examined the effect of the ChAdOx1 nCoV-19 vaccine on the frequencies of the total monocyte (CD14^^+^^) population and nonclassical/intermediate monocytes that coexpress CD16 (CD14^^+^^CD16^^+^^), which exert effector function in humans during infection and inflammation (19, 20). In addition to being a marker for monocytes, CD14 is as a coreceptor for TLR4 and facilitates cellular responses to LPS or Gram-negative bacteria (21). Cell surface–bound CD14 is cleaved and released as a scavenger receptor when monocytes are activated (22). Median fluorescence intensity (MFI), a surrogate for relative protein expression, of CD14 was significantly decreased on day 14 after vaccination but returned to baseline thereafter (Figure 2B). This may be indicative of monocyte activation, suggesting that there is prolonged innate immune activation 14 days after vaccination, which returns to homeostasis by day 56. The absolute number of monocytes was determined by multiplying the cell count obtained from the Percoll monocyte enrichment process by the frequency of CD14^^+^^ cells present in the enriched population, as determined by flow cytometry (Figure 2C). The monocyte frequency in the blood was calculated by dividing the absolute number of monocytes by the absolute number of PBMCs (Figure 2D). The absolute numbers (Figure 2C) and the frequencies (Figure 2D) of monocytes in the peripheral blood were significantly increased at all time points after vaccination compared with prevaccine controls. The absolute numbers and frequencies of monocytes coexpressing CD14 and CD16 was significantly increased at all time points after vaccination compared with prevaccine controls (Figure 2, E and F). Cumulatively, these data suggest that myelopoiesis is enhanced after vaccination and maintained for up to 3 months.

### Increased expression of antigen presentation molecules on monocytes was sustained for up to 3 months after vaccination with ChAdOx1 nCoV-19.

Antigen presentation by innate cells to activate the adaptive immune system is a crucial event in the vaccination process. In animal models, trained monocytes show an increased expression of MHC-II and cell costimulatory molecules such as CD80 and CD86 (13, 23). Human dendritic cells matured with β-glucan, a fungal cell wall component known to induce trained immunity, were shown to have enhanced HLA-DR, CD40, CD80, and CD86 expression (24). Furthermore, human monocytes trained with *C*. *albicans* had increased RNA levels of *HLA-DRB1*, *CD40*, *CD80*, and *CD86* (25). There is evidence to suggest that trained cells may enhance T cells responses (26); therefore, we sought to determine the effect of the ChAdOx1 nCoV-19 vaccine on antigen-presenting functions of monocytes.

**Figure 3:**
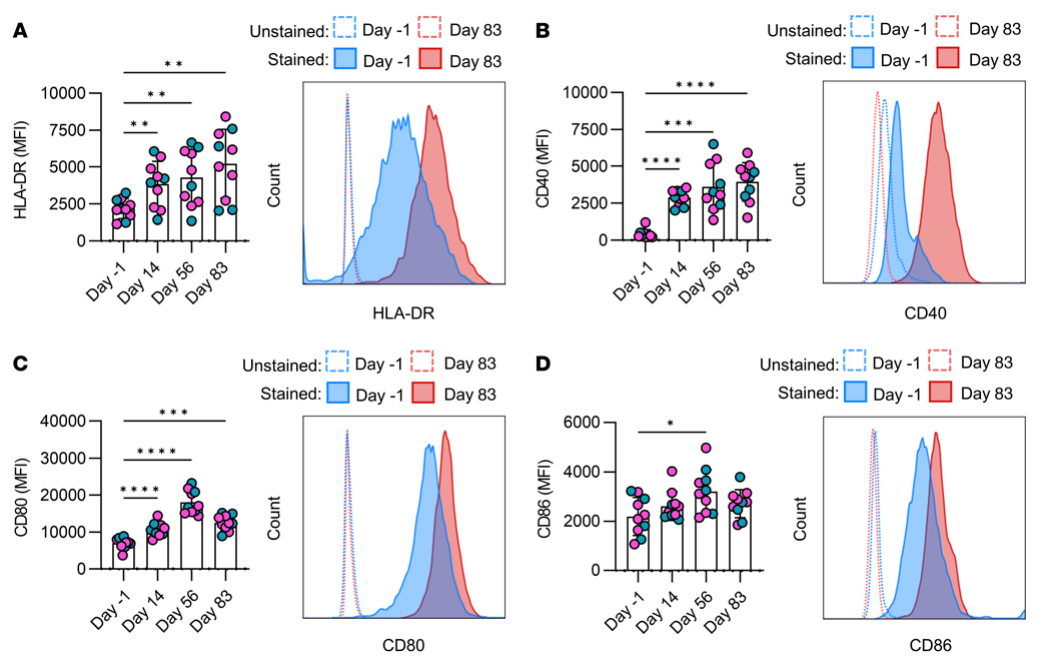
Vaccination enhanced the expression of cell markers associated with antigen presentation and T cell activation. Monocytes were enriched from healthy donor PBMCs on day –1 (prevaccine), day 14, day 56, and day 83 after vaccination using a hyperosmotic Percoll gradient. The cell surface expression of (**A**) the antigen presentation marker HLA-DR and (**B**–**D**) the T cell costimulatory molecules (**B**) CD40, (**C**) CD80, and (**D**) CD86 on ex vivo monocytes was assessed by flow cytometry. Graphs show collated data, with each dot representing an individual donor (*n* = 10); blue dots denote male donors, and pink dots denote female donors. Representative histograms illustrate the difference in the median fluorescence intensity of each marker in stained (full outline) and unstained (dotted outline) samples on day –1 (blue) and day 83 (red). Data are graphed as the mean value ± SD. Statistically significant differences between the groups were determined by a repeated measures 1-way ANOVA using Tukey’s multiple comparisons test; *P < 0.05, **P < 0.01, ***P < 0.001, ****P < 0.0001.

PBMCs were isolated from the blood at the time points previously indicated (Figure 1A), and monocytes were enriched using a hyperosmotic Percoll gradient. Cells were Fc blocked and stained with fluorochrome-conjugated antibodies specific for CD14, CD68, CD40, CD80, HLA-DR, and CD86 prior to acquisition by flow cytometry. Expression of HLA-DR, CD40, and CD80 on CD14^^+^^ monocytes was significantly increased on days 14, 56, and 83 after vaccination (Figure 3, A–C), whereas CD86 expression was only significantly increased on day 56 after vaccination (Figure 3D) compared with prevaccine controls (day –1). Representative histograms show the staining for each marker on day –1 and day 83 compared with their unstained controls. Monocyte subpopulations were analyzed by gating on CD14^^+^^CD16^^–^^ classical monocytes, CD14^^+^^CD16^^+^^ intermediate monocytes, and CD14^^dim^^CD16^^+^^ nonclassical monocytes (Supplemental Figure 1; supplemental material available online with this article; https://doi.org/10.1172/JCI162581DS1). All subpopulations exhibited statistically significantly increased expression of the surface markers examined, recapitulating the overall effects observed in the total population. These data indicate that the ChAdOx1 nCoV-19 vaccine enhances the antigen-presenting ability of monocytes for up to 3 months after vaccination.

### Monocytes exhibit enhanced cellular energetics up to 2 months after vaccination.

**Figure 4:**
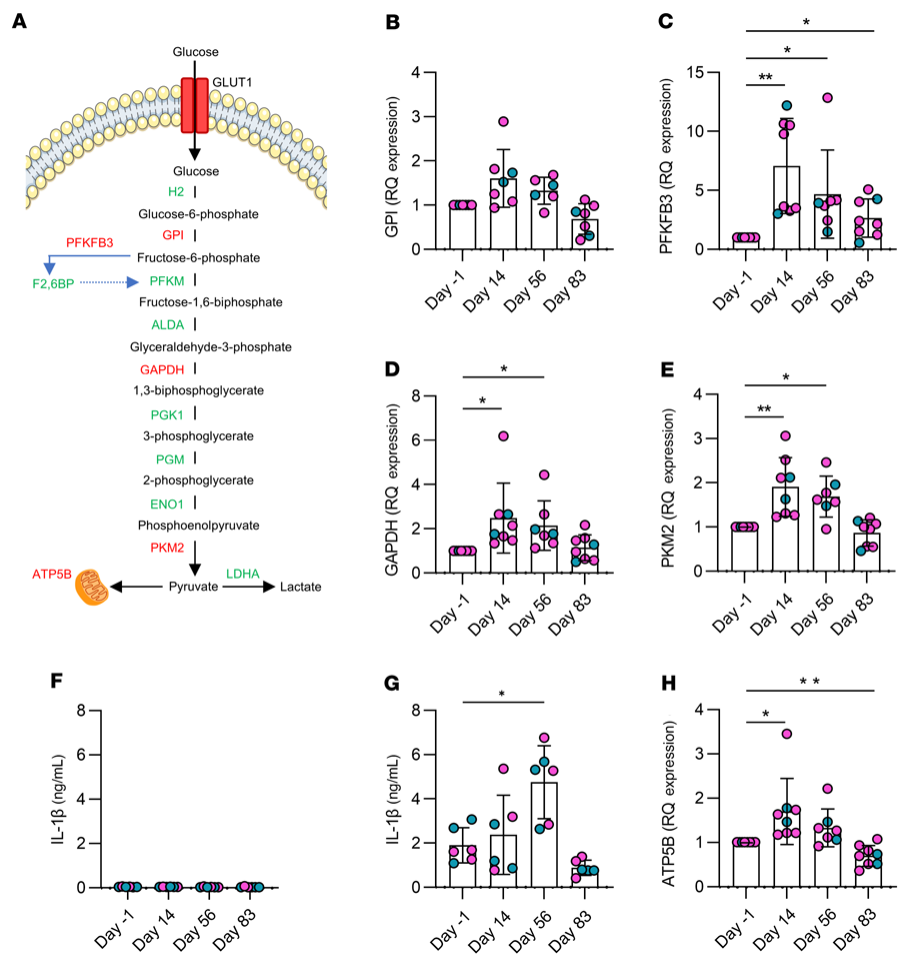
Monocytes were metabolically reprogrammed after vaccination. (**A**) A diagram showing the breakdown of glucose to pyruvate via the glycolytic pathway. Pathway intermediates are shown in black, enzymes are shown in green, and the enzymes analyzed in this study are shown in red. Monocytes were enriched from healthy donor PBMCs on the day before (day –1) and days 14, 56, and 83 after vaccination using a hyperosmotic Percoll gradient. (**B**–**E**) Relative expression of transcript levels of (**B**) *GPI*, (**C**) *PFKFB3*, (**D**) *GAPDH*, and (**E**) *PKM2* are shown. (**F** and **G**) Isolated monocytes were stimulated ex vivo with (**F**) medium or (**G**) irradiated *M*. *tuberculosis* (10 μg/mL iH37Rv), and the concentration of IL-1β in the supernatant was measured by multiplex ELISA. (**H**) Relative expression of transcript levels of *ATP5B*, a gene marker of oxidative phosphorylation, was also determined. Gene expression was determined using RT-qPCR. Each dot represents an individual donor (*n* = 6–8), with blue dots denoting male donors and pink dots denoting female donors. Statistically significant differences between the groups were determined by (**B**–**E** and **H**) a mixed-effects model (REML) ANOVA, with Šídák’s multiple comparisons test and (**G**) a repeated measures 1-way ANOVA using Dunnett’s multiple comparisons test; *P < 0.05, **P < 0.01.

Both in vitro and in vivo studies have shown that metabolic reprogramming is an essential event in the induction of trained immunity, which is mechanistically associated with enhanced protection against unrelated pathogens (10, 27–31). We sought to determine if the ChAdOx1 nCoV-19 vaccine effected the cellular metabolism of monocytes over time. Therefore, we examined the transcript expression levels of key glycolytic enzymes, *GPI*, *PFKFB3*, *GAPDH*, and *PKM2*, by RT-qPCR. These genes encode enzymatic proteins throughout the glycolytic pathway (Figure 4A).

Vaccination did not significantly alter the expression of *GPI* in monocytes (Figure 4B); however, expression of *PFKFB3* was significantly increased on days 14, 56, and 83 after vaccination compared with prevaccine controls (Figure 4C). *GAPDH* and *PKM2* were significantly increased on day 14 and 56 after vaccination but had returned to prevaccination levels by day 83 (Figure 4, D and E).

Because the production of IL-1β is associated in the literature with enhanced glycolysis (32–34), we sought to determine if the enhanced expression of glycolytic enzymes in monocytes after vaccination resulted in enhanced production of IL-1β. In addition, trained immunity has been shown to increase the ability of myeloid cells to produce IL-1β. For example, human monocytes trained with β-glucan in vitro increased the concentration of intracellular IL-1β in response to *M*. *tuberculosis* stimulation, and murine bone marrow–derived macrophages from BCG-vaccinated mice showed increased expression of *IL1B* (5, 17). This is likely due to the increase in glycolysis induced by trained immunity (10, 30). Furthermore, airway macrophages from mice vaccinated intranasally with an adenovirus-vectored *M*. *tuberculosis* vaccine produced significantly more IL-1β in response to LPS and *M*. *tuberculosis* whole cell lysate compared with unvaccinated mice (14).

Monocytes were isolated and stimulated ex vivo with irradiated *M*. *tuberculosis* (iH37Rv) for 24 hours. The concentration of IL-1β was determined by multiplex ELISA (Figure 4, F and G). There was no difference in the detection of IL-1β in unstimulated cells over time (Figure 4F). Monocytes stimulated with irradiated *M*. *tuberculosis* produced significantly more IL-1β on day 56 after vaccination compared with their prevaccine controls (Figure 4G).

To determine if metabolic reprogramming was restricted to glycolysis, or if vaccination could affect oxidative phosphorylation, we quantified the expression of *ATB5B* (a gene that encodes ATP synthase and is used as a marker of oxidative phosphorylation; Figure 4H) in monocytes before and after vaccination. Expression of *ATB5B* was significantly increased on day 14 and significantly decreased by day 83 after vaccination compared with prevaccine controls (Figure 4H).

Taken together, these data indicate that monocytes are metabolically reprogrammed toward enhanced glycolysis for 2 months in people vaccinated with a single dose of the ChAdOx1 nCoV-19 vaccine. Moreover, these reprogrammed monocytes have increased production of the key proinflammatory cytokine, IL-1β, at day 56 after vaccination compared with prevaccine controls, demonstrating that these cells have increased functional outputs that have previously been associated with enhanced capacity for glycolysis.

### The production of cytokines and chemokines is altered in monocytes following vaccination.

**Figure 5:**
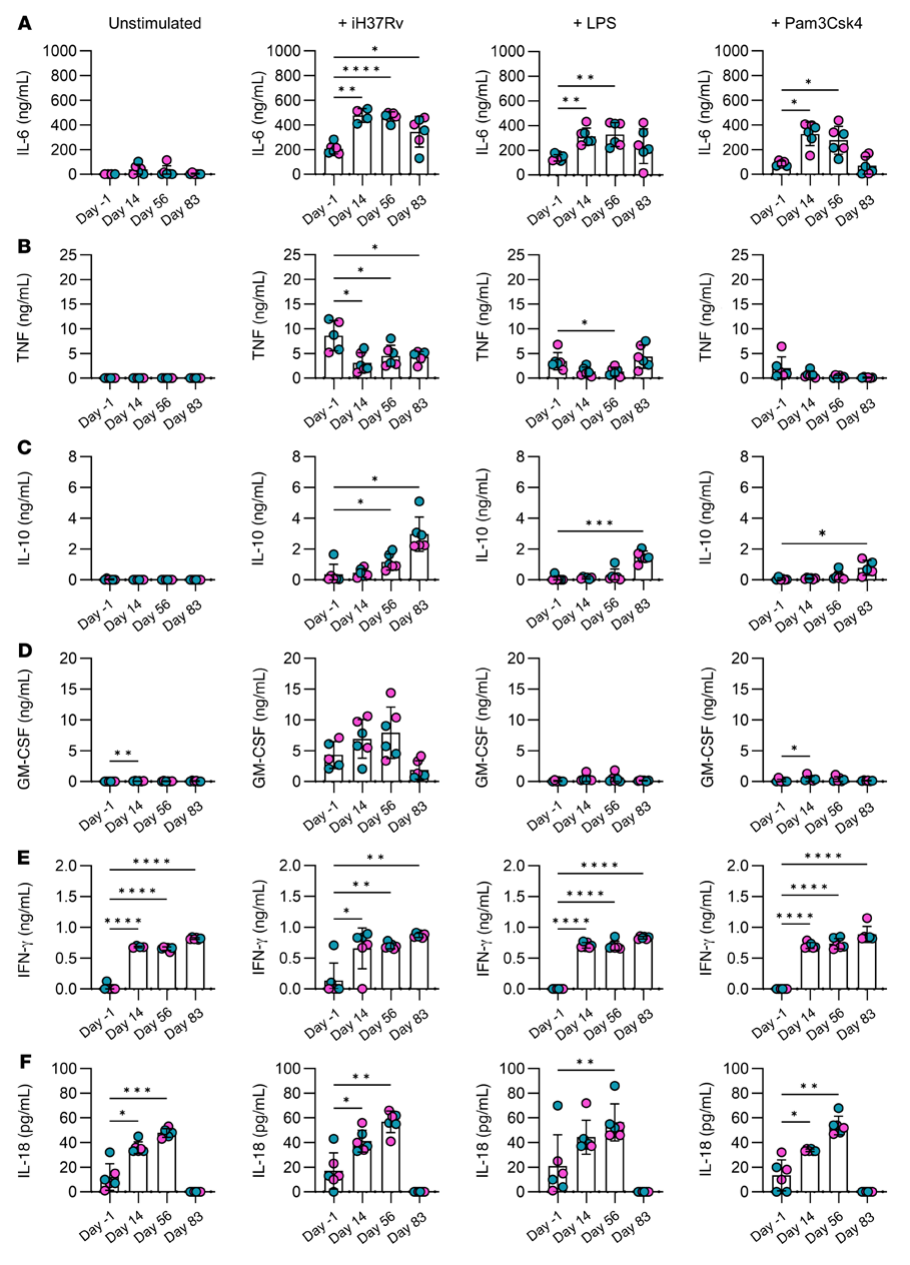
Vaccination altered cytokine production in response to unrelated stimuli. Monocytes were isolated from healthy donor PBMCs on the day before (day –1) and days 14, 56, and 83 after vaccination using a hyperosmotic Percoll gradient. Monocytes were further purified using plastic adherence and were routinely over 90% pure. Monocytes were left to rest overnight and stimulated ex vivo with medium (unstimulated), irradiated *M*. *tuberculosis* (iH37Rv; 10 μg/mL), LPS (10 ng/mL), or Pam3Csk4 (10 μg/mL) for 24 hours. The concentrations of (**A**) IL-6, (**B**) TNF, (**C**) IL-10, (**D**) GM-CSF, (**E**) IFN-γ, and (**F**) IL-18 in the supernatants were assessed using a multiplex ELISA, with **A**–**E** showing ng/mL and **F** showing pg/mL. Each dot represents an individual donor (*n* = 6), with blue dots denoting male donors and pink dots denoting female donors. Data are graphed as the mean value ± SD. Statistically significant differences between the groups were determined by a repeated measures 1-way ANOVA using Dunnett’s multiple comparisons test; *P < 0.05, **P < 0.01, ***P < 0.001, ****P < 0.0001.

**Figure 6:**
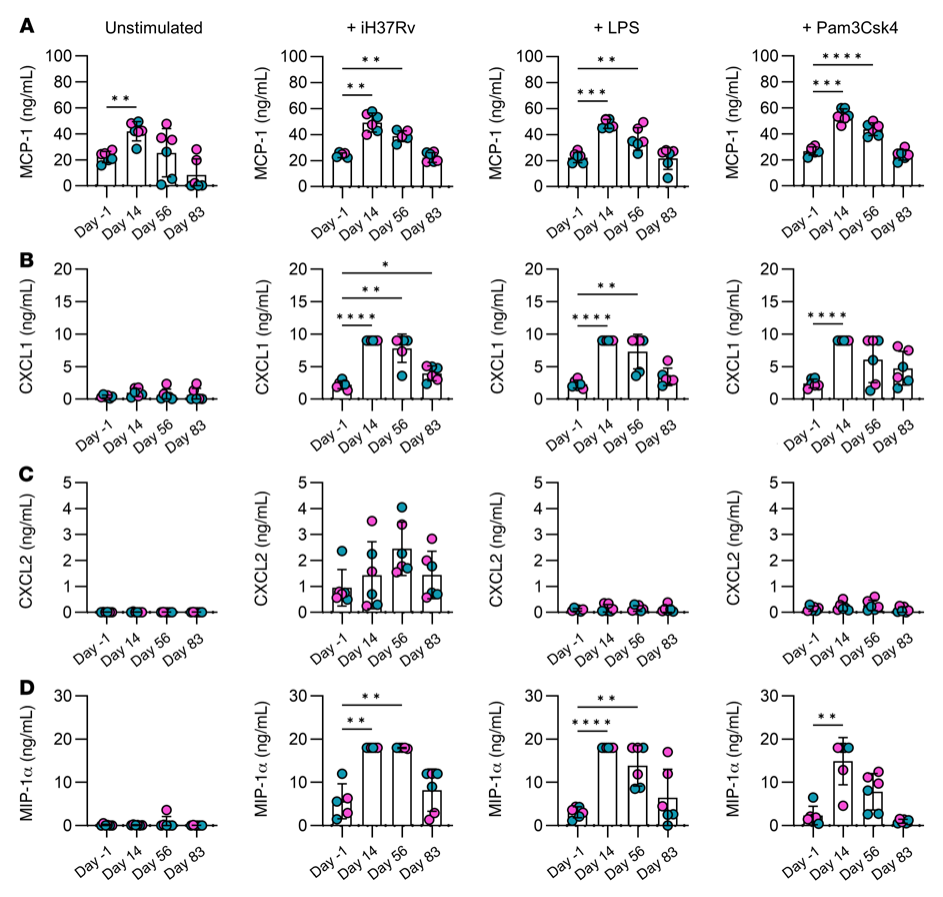
Vaccination results in altered chemokine production in response to unrelated stimuli. Monocytes were enriched from healthy donor PBMCs on the day before (day –1) and days 14, 56, and 83 after vaccination using a hyperosmotic Percoll gradient. Monocytes were further purified using plastic adherence and were routinely over 90% pure. Monocytes were left to rest overnight and stimulated ex vivo with medium (unstimulated), irradiated *M*. *tuberculosis* (iH37Rv; 10 μg/mL), LPS (10 ng/mL), or Pam3Csk4 (10 μg/mL) for 24 hours. The concentrations of (**A**) MCP-1, (**B**) CXCL1, (**C**) CXCL2, and (**D**) MIP-1α in ng/mL in the supernatants were assessed using a multiplex ELISA. Graphs show collated data, with each dot representing an individual donor (*n* = 6) and blue dots denoting male donors and pink dots denoting female donors. Data are graphed as the mean value ± SD. Statistically significant differences between the groups were determined by a repeated measures 1-way ANOVA using Dunnett’s multiple comparisons test; *P < 0.05, **P < 0.01, ***P < 0.001, ****P < 0.0001.

The effects of trained immunity resulting in nonspecific protection against infection are mediated by enhanced production of cytokines and chemokines that expedite and amplify the subsequent immune response (2, 4, 13, 17). Monocytes were enriched from PBMCs using a Percoll gradient and further purified by adherence to plastic for 1 hour. Cells were stimulated ex vivo with iH37Rv, TLR4 agonist LPS, or TLR2 agonist Pam3Csk4 for 24 hours. The concentrations of cytokines, IL-6, TNF, IL-10, GM-CSF, IFN-γ, IL-18, IL-4, IL-8, IL-12p70, IL-23, and IFNα2a (Figure 5 and Supplemental Figure 2), and chemokines, CXCL1, CXCL2, MIP-1α, and MCP-1 (Figure 6), were determined by Assay Genie Multiplex ELISA.

IL-6 is a pleiotropic cytokine with a critical role in the induction of inflammation, including acting as a pyrogen. As such, it is an early mediator of the inflammatory response to a diverse range of insults, including bacterial and viral infections. Production of IL-6 was previously shown to be enhanced in monocytes that exhibit trained immunity (2, 4, 17); therefore, we examined the production of IL-6 in response to stimulation in monocytes from people who had undergone vaccination compared with their prevaccine control monocytes (Figure 5A). Monocytes stimulated with irradiated *M*. *tuberculosis* had significantly increased production of IL-6 at all time points up to 3 months (83 days) after vaccination compared with prevaccine controls. Similarly, monocytes stimulated with TLR agonists LPS or Pam3Csk4 produced significantly more IL-6 than their prevaccine controls at day 14 and day 56 after vaccination (Figure 5A). Enhanced TNF is also associated in the literature with trained immunity induced by BCG or β-glucan (2, 4, 17) and is known to be seminal in the response to *M*. *tuberculosis* infection (35). Interestingly, our data indicated that production of TNF in response to stimulation with *M*. *tuberculosis* was significantly decreased after vaccination (Figure 5B). In addition, cells stimulated ex vivo with LPS exhibited significantly reduced production of TNF on day 56 after vaccination compared with the prevaccine control, and this was restored by day 83 after vaccine. The antiinflammatory cytokine IL-10 was significantly increased in response to *M*. *tuberculosis* stimulation in monocytes at day 56 and day 83 after vaccination; this was recapitulated in monocytes stimulated with LPS or Pam3Csk4 on day 83 (Figure 5C). GM-CSF was significantly increased on day 14 after vaccination in resting cells and in cells stimulated with Pam3Csk4 but was not significantly different in *M*. *tuberculosis*– or LPS-treated cells over time (Figure 5D). Strikingly, IFN-γ was significantly increased in resting cell cultures on days 14, 56, and 83 after vaccination compared with the prevaccine control (Figure 5E). Similarly, the concentrations of IL-18 present in the supernatants were significantly increased in unstimulated cells on day 14 and day 56 after vaccination compared with prevaccine controls (Figure 5F). Concentrations of IL-4 and IL-8 were not significantly changed over time or with stimulation (Supplemental Figure 2, A and B). Production of IL-12p70 was significantly decreased over time in monocytes stimulated with *M*. *tuberculosis* or Pam3Csk4; however, the overall concentrations detected were very low (Supplemental Figure 2C). Detection of IFNα2a was also very low; however, monocytes stimulated with *M*. *tuberculosis* on day 56 after vaccination had significantly increased production of IFNα2a compared with day –1 (Supplemental Figure 2D). IL-23 production was significantly increased on day 14 compared with day –1 in response to *M*. *tuberculosis* stimulation (Supplemental Figure 2E).

Next, we analyzed the concentrations of key chemokines produced by monocytes prior to vaccination compared with days 14, 56, and 83 after vaccination (Figure 6). Mice trained with adenovirus vector were protected against *S*. *pneumoniae* owing to faster induction of MCP-1, KC (also known as CXCL1 in humans), MIP-1α (also known as CCL3), and MIP-2 (also known as CXCL2), leading to increased early recruitment of neutrophils, which mediated bacterial clearance in the lung (13). Our data indicate that the concentrations of MCP-1 are significantly increased in resting monocytes 14 days after vaccination compared with prevaccine controls (Figure 6A). When cells were stimulated with *M*. *tuberculosis*, LPS, or Pam3Csk4, the concentrations of MCP-1 produced were significantly increased on day 56 after vaccination compared with day –1. Production of CXCL1 was significantly increased in response to *M*. *tuberculosis* stimulation on days 14, 56, and 83 after vaccine compared with day –1. CXCL1 was similarly significantly increased in response to LPS stimulation on day 14 and 56, whereas it was only significantly increased on day 14 in cells stimulated ex vivo with Pam3Csk4 (Figure 6B). Production of CXCL2 was not significantly altered over time in any stimulation group (Figure 6C). MIP-1α was significantly enhanced on day 14 and day 56 in response to *M*. *tuberculosis* or LPS stimulation compared with prevaccine controls but only significantly increased on day 14 after vaccination in cells stimulated with Pam3Csk4 (Figure 6D).

Taken together, these data strongly indicate that monocyte function was altered on day 14, day 56, and up to day 83 after vaccination. Cells showed enhanced capacity to produce IL-6, CXCL1, and MIP-1α in response to unrelated stimuli. Moreover, resting cells in culture ex vivo for 24 hours produced significantly increased concentrations of IFN-γ, IL-18, and MCP-1 after vaccination compared with the prevaccine controls.

## Discussion

These data indicate that the ChAdOx1 nCoV-19 vaccine induced trained immunity in humans in vivo. Together with evidence indicating that adenovirus vector–based, but not mRNA-based, COVID-19 vaccines may exert nonspecific protective effects in humans (12) and that empty adenovirus vector can induce trained immunity in mice (13), we postulate that the effects observed may be caused, at least in part, by the vector rather than the payload. In support of this, intranasal empty adenovirus vector vaccine and adenovirus vectors loaded with *M*. *tuberculosis* or SARS-CoV-2 antigens induced trained immunity in airway macrophages identified by their high expression of MHC-II and increased glycolytic metabolism (13, 14, 36), similar to the phenotype we observed in peripheral blood monocytes after intramuscular vaccination. Our data demonstrate that monocyte absolute numbers and frequencies were increased in the blood up to 83 days after vaccination, indicative of a preferential skewing of hematopoiesis toward myelopoiesis, which is associated with the induction of trained immunity in animal models (11, 16, 37) and in humans (18). Notably, the fluorescence intensity, which is directly correlated with the cell surface expression level, of CD14 was decreased on day 14 after vaccination. This decrease in the cell surface CD14 protein is associated with monocyte activation (22) and, therefore, suggests that, at this early time point, there is evidence of prolonged innate immune activation, even though the initially stimulated innate response should have resolved by day 14 after vaccination. The return to baseline of CD14 expression by day 56 may be indicative of the cells returning to homeostasis.

Elevated expression of HLA-DR on monocytes in vivo after vaccination closely recapitulates the increased MHC-II expression in tissue-resident macrophages in the mouse model of trained immunity induced by an adenovirus vector (13, 14). In contrast, gene expression of HLA-DR was downregulated in people 2 weeks and 3 months after BCG vaccination compared with the prevaccine control (38), suggesting that the features of trained innate immunity may differ according to the inducing stimulus. We observed increased expression of costimulatory molecules CD40, CD80, and CD86 after vaccination. However, CD80 and CD86 were not enhanced in macrophages from the lungs of mice exposed to the adenovirus vector (13). This divergence may be due to human versus mouse variation or due to tissue-specific versus peripherally induced trained immunity. When we segregated the data into monocyte subpopulations, statistically significant upregulation of HLA-DR and costimulatory molecule expression was evident in all subpopulations, suggesting that this feature of trained innate immunity may occur in the precursor stem cells and not because of activation of the mature monocyte.

When we examined the relative expression of key glycolytic enzymes by RT-qRT-PCR, we observed significantly increased *PFKFB3* at all time points after vaccination, whereas *GAPDH* and *PKM2* were significantly increased on day 14 and day 56 after vaccination and trended back down toward prevaccine control levels by day 83. This indicates that the cells exposed to the adenovirus vector–based vaccine have increased capacity to undergo glycolysis up to 2 months after vaccination and this is in keeping with the established role for glycolysis in the induction of trained immunity in response to BCG vaccination or β-glucan (10, 30). Expression of mitochondrial *ATP5B*, a marker of cell capacity to carry out oxidative phosphorylation, was significantly increased on day 14 and significantly decreased on day 83, indicating that the cells had enhanced capacity for both glycolysis and oxidative phosphorylation on day 14 after vaccination. The role of oxidative phosphorylation in the induction of trained immunity and the ensuing immunometabolic response of the trained cells upon restimulation is not yet fully known, and there are divergent conclusions, which are dependent on the dose of the training stimulus and the model (39). Our data show that monocytes in vivo have increased capacity for glycolysis up to 3 months after vaccination, indicative of metabolic reprogramming, akin to that observed during trained immunity induced by BCG vaccination or by β-glucan (39). Furthermore, our data demonstrating enhanced production of IL-1β in response to stimulation ex vivo with *M*. *tuberculosis* after vaccination suggests that this glycolytic reprogramming may have a functional effect on cytokine production, because production of IL-1β has been previously associated with increased flux through glycolysis in the literature (32, 33, 40–43).

When monocytes were stimulated with unrelated antigens (whole irradiated *M*. *tuberculosis*, LPS, or Pam3Csk4), the production of IL-6 was significantly increased after vaccination compared with prevaccine controls. Conversely, production of TNF was significantly reduced at all time points after vaccination in response to *M*. *tuberculosis* stimulation and at day 56 in LPS-stimulated cells. This contrasts with the effects of trained innate immunity induced by BCG vaccination, intranasal adenovirus vector vaccination, or by β-glucan, which are reported to increase TNF production upon secondary stimulation (2, 14, 17). However, trained immunity induced by an extract from *Fasciola hepatica* decreased production of TNF in macrophages stimulated with LPS (44). It is therefore plausible that the cytokine profile induced by trained immunity differs depending on the microbial stimulus, and further work to define the distinct features of trained innate immunity induced by different types of vaccines or microbial exposures is required. Another plausible explanation may be that trained immunity induced by ChAdOx1 nCoV-19 vaccination may increase the expression of TNF receptors on the monocytes and they may, therefore, have increased capacity to take up TNF in an autocrine manner at the after vaccination time points. This may explain why the accumulation of TNF was reduced 24 hours after stimulation in the supernatants of monocytes after vaccination.

Interestingly, in cells stimulated with a single TLR agonist (either LPS or PamCsk4), the increase in IL-6 production was only evident at day 14 and day 56 after vaccination, indicating that by day 83 the effect of trained immunity may be waning. This trend was observed in other data sets too, including the expression of glycolytic enzymes and the increased production of IL-1β and chemokines MCP-1, CXCL1, and MIP-1α. Cumulatively, these data indicate that by day 83 after vaccination, the phenotype and function of the monocytes may be changing. Conversely, at day 83 after vaccination, IL-10 was significantly increased compared with prevaccine controls. Monocytes frequencies, numbers, and their expression of antigen presentation and costimulatory molecules were stably increased up to and including day 83.

MCP-1, GM-CSF, and expression of *ATP5B* were significantly increased in resting monocytes on day 14 after vaccination but not at later time points. Given that the expression intensity of CD14 on the surface of monocytes was reduced at day 14 compared with all other time points, this indicates that the cells may still be activated from the initial insult from the vaccine. The data may indicate that prolonged innate immune activation occurred 2 weeks after vaccination. Furthermore, production of IL-1β in response to stimulation with *M*. *tuberculosis* was significantly increased at day 56 but not at day 14, suggesting that trained immunity may not yet be induced at this earlier time point. By 2 months (56 days), the cells exhibited features consistent with trained immunity, and then by 3 months (83 days) after vaccination, this effect was waning in some of the data sets. However, many of the phenotypes were maintained at day 83, so it is plausible that the effects of trained innate immunity are longer lasting but could not be analyzed in this study without the confounding factor of the booster dose. Longitudinal studies using single-dose adenoviral vector vaccines are therefore warranted to assess the longevity of these effects on the innate immune responses.

Strikingly, resting monocytes taken from donors 14 and 56 days after vaccination produced IFN-γ, IL-18, and MCP-1 in culture without stimulation. It is difficult to conclude whether this is in keeping with monocytes that have undergone trained immunity, because much of the published data present changes in cytokine production as fold change or do not show the complete data set with unstimulated controls (2–4, 17). However, trained immunity induced in human monocytes in vitro by β-glucan resulted in elevated gene expression of MCP-1 and CCL18 in resting cells on day 6 after training, which was then further elevated upon restimulation with *M*. *tuberculosis* (17). In addition, the significantly elevated GM-CSF observed in resting monocytes on day 14 after vaccination may also play a key role in the enhancement of myelopoiesis and reprogramming toward trained immunity, in keeping with its role in inducing trained immunity in the bone marrow of mice exposed to β-glucan (11). The increased production of IL-18 observed in resting monocytes, in addition to the increased IFN-γ, suggests that NK cell function and activation may be enhanced in this setting (45, 46). Moreover, features of trained immunity can be induced in NK cells by BCG vaccination, viral infection, or exposure to cytokines in vitro, including IL-18 (47–50). We, therefore, postulate that NK cells may play a key role in trained immunity induced by ChAdOx1 nCoV-19 vaccination, and further studies are warranted to determine this.

While these elevated proinflammatory cytokines may be beneficial in mediating nonspecific immunity against other infections, it is also plausible that this inflammation may be deleterious in certain contexts. For example, MCP-1 (also known as CCL2) regulates the migration of monocytes, and other immune cells, from the blood into the tissue. It has previously been associated with inflammatory atherosclerosis, which is mediated, at least in part, by trained immunity (29, 51). However, our data indicate that the ability to produce regulatory IL-10 increases over time, which may protect against excessive inflammation.

Our data indicate that intramuscular delivery of the ChAdOx1 nCoV-19 vaccine can induce trained immunity in the peripheral blood. We do not know whether these monocytes with enhanced function would exert expedited and elevated responses to subsequent infections in tissues such as the lung. Work in animal models shows that intranasally delivered adenoviral vaccine vector can induce trained immunity in tissue-resident alveolar macrophages, which results in enhanced protection against a subsequent challenge (13, 14, 36). This tissue-specific trained immunity was mediated by IFN-γ (13). Further evidence in BCG-treated mice indicates that IFN-γ is required to induce trained immunity in the bone marrow hematopoietic stem cell niche (5). In keeping, IFN-γ is required for the induction of trained immunity in human monocytes stimulated with BCG (52). Taken together, these data provide a strong rationale, suggesting that the elevated IFN-γ observed ex vivo in human monocytes after ChAdOx1 nCoV-19 vaccination may mechanistically mediate the increased myelopoiesis and the effects of trained immunity observed herein. In addition, metabolic reprogramming induced by ChAdOx1 nCoV-19 vaccination is likely to result in epigenetic changes that mediate the enhanced monocyte function in response to subsequent unrelated stimuli (53).

Trained immunity induced by BCG is dependent on NOD-2 in vivo in humans (2). IL-6 and MCP-1 produced in response to adenovirus vectors are significantly reduced in NOD-2 deficient mice (54). Furthermore, adenovirus synergizes with the NOD-2 agonist muramyl dipeptide, resulting in increased IL-1β and TNF production (55). Therefore, we postulate that NOD-2 may have a role in mediating trained innate immunity induced by the ChAdOx1 nCoV-19 vaccine.

Because downstream responses to both TLR2 and TLR4 agonists are enhanced after ChAdOx1 nCoV-19 vaccination, we speculate that common signaling molecules in these pathways may be mechanistically involved in the induction of trained immunity, such as MyD88, NF-κB, and activation of NLRP3 (required for the caspase-dependent processing of IL-1β and IL-18, both of which are increased in our data). In summary, we suggest that the mechanisms underpinning the trained innate immunity elicited by ChAdOx1 nCoV-19 vaccination may include a role for IFN-γ, epigenetic reprogramming, NOD-2, and innate signaling transducers.

Our data may aid in the design of novel vaccination strategies that combine traditional intramuscular routes with intranasal/airway delivery of the vaccine. This may result in trained immunity in circulating monocytes and other myeloid cells, which may have enhanced efficacy against respiratory infection in combination with trained tissue-resident alveolar macrophages. Because both the alveolar macrophage and the infiltrating monocyte-derived macrophage have distinct but important roles to play during infection, supporting the functions of both populations may have increased benefit in host defense. In addition, understanding the kinetics of enhanced innate immune function after the priming dose of vaccination may be crucial to optimize the timing of subsequent booster doses. Real-world evidence from the clinical trials for ChAdOx1 nCoV-19 indicates that the booster dose induced better efficacy when administered more than 8 weeks after priming compared with boosters given less than 6 weeks after the initial immunization (56). The molecular mechanism behind this observation is unknown; however, there is evidence indicating that kinetics of germinal center formation and increased selection of B cells with higher antigen affinity occur when the boosting time frame is delayed (57). The increased antigen-presenting function and cytokine/chemokine profile we observed is likely to enhance adaptive memory responses to booster vaccination. Our data indicate that prolonged innate immune activation occurs at day 14 (2 weeks) and then changes to a phenotype more consistent with trained innate immunity by day 56 (8 weeks). Therefore, we postulate that the kinetics of innate immune function may be a significant contributor to the improved vaccine booster efficacy observed after 8 weeks compared with boosters given earlier than 6 weeks after priming. We, and others (58), propose that tracking innate immune responses in addition to traditional B and T cell responses after the priming vaccination may therefore help to identify the optimal vaccine regimen.

Our data suggest that trained immunity may be induced in humans by other vaccines using similar adenovirus vector-based platforms being developed in murine models for TB and next-generation COVID-19 vaccines (14, 36). However, we postulate that trained innate immunity induced by distinct adenoviral vectors with different target antigens will likely induce differential innate immune profiles. Therefore, further investigations are warranted to enable us to specifically understand the types of trained innate immunity induced by discrete immune stimuli and the subsequently elicited protective versus potential pathogenic effects to allow us to harness the potential of trained innate immunity toward clinical benefit.

### Study limitations.

While our study provides evidence that the ChAdOx1 nCoV-19 vaccine induced phenotypic and functional changes in myeloid cells, consistent with those previously reported in the literature in other settings of trained immunity, we did not have the capacity to undertake epigenetic profiling of monocytes before and after vaccination.

We cannot definitively conclude if this altered monocyte function in response to vaccination was caused by the adenovirus vector or the SARS-CoV2 spike protein payload or a combination of both. However, there is evidence in the literature to suggest that empty adenovirus vectors can induce trained immunity (13), which allows us to postulate that the vector likely plays a key role in the induction of trained immunity in our study. Moreover, because we examined the effects on monocytes in a naive population up to 3 months after the single, initial vaccine dose (and prior to the booster), it is unlikely that our results are confounded by an adaptive memory response.

Because exposure to microbes induces trained immunity, the study of these effects in a human population is confounded owing to continuous environmental exposure to pathogens. Despite our small cohort, our data illustrated statistically significant effects, indicating that this study was appropriately powered. In addition, our study design also benefits from the longitudinal nature of the sample collection, whereby every donor is their own control (before and after vaccine). We began our study in Dublin, Ireland, in early March 2021 (day –1) during a period of extended social restrictions (from late December 2020 to June 2021). All other blood draws also occurred within this period (day 83 was drawn at the end of May 2021). Therefore, the probability of our donors being exposed to SARS-CoV2 (or other infections) is likely to be lower than that expected normally during longitudinal studies; however, we cannot definitively rule out the occurrence of an asymptomatic infection in our donors. The Health Protection Surveillance Centre indicates that during that period, from available data, there were very low rates of influenza and RSV, suggesting that the social restrictions may have reduced the background confounding variables of exposure to other infections in a human population (59).

We cannot rule out that seasonal variation may contribute somewhat to the changing immune phenotypes observed over time in our study. However, 5 of 10 of our volunteers took regular multivitamin supplements, with 3 of these 5 donors specifically taking vitamin D supplements. We did not observe substantial spread in any of our data that may indicate that seasonal variation was mitigated with vitamin D supplementation.

Trained innate immunity and heterologous effects of vaccines have a sex differential (60, 61). Although 4 male and 6 female participants were included in the study, when we segregated the data based on sex, we did not find any statistically significant differences (data not shown); however, we acknowledge that our study was not sufficiently powered to determine a sex differential. For transparency we have color coded each data point to differentiate between male and female donors throughout the data sets.

### Conclusion.

The ChAdOx1 nCoV-19 vaccination induced prolonged innate immune activation, with evidence to support the hypothesis that adenoviral vector–based vaccines induce trained immunity in humans. Our study is the first, to our knowledge to show that monocyte phenotype and proinflammatory function both at baseline and in response to subsequent unrelated insults, is enhanced up to 3 months after vaccination with a single dose of the ChAdOx1 nCoV-19 vaccine. These data improve our understanding of the contributions of innate immune responses to vaccine efficacy and to heterologous vaccine effects and may aid in the design of future vaccines or innovative vaccine strategies.

## Methods

### Monocyte isolation.

This study used venous blood from healthy volunteers (*n* = 10) who were aged between 23 and 35 years. Exclusion criteria for blood donation at all time points included if a volunteer had received any other vaccines in the last 3 months, anyone who had ever previously tested positive for COVID-19, or a participant feeling unwell on the day of blood donation. The volunteer cohort included 6 female and 4 male participants. Four donors declared taking daily medication (none of which were specifically immunomodulatory): levothyroxine, escitalopram, and norethindrone. Venous blood was drawn on the day before the first dose of the ChAdOx1 nCoV-19 vaccine (day –1) and at days 14, 56, and 83 after vaccination. PBMCs were isolated from the blood by density-gradient centrifugation over Lymphoprep (StemCell Technologies, catalog 07861). Cells were then washed in RPMI (Gibco, catalog 61870036). Monocytes were separated by hyperosmotic density gradient centrifugation over Percoll (GE Healthcare, catalog 17-0891-01). Following isolation, PBMCs were layered on top of a hyperosmotic Percoll solution (48.5% Percoll, 41.5% sterile H2O, 0.16 M NaCl) and centrifuged for 15 minutes at 580*g*. The interphase layer was isolated, and cells were washed with cold PBS and resuspended at a concentration of 1 × 10^^6^^ cells/mL.

For experiments where *n* < 10, omitted donors had insufficient cell numbers or RNA yields to carry out the complete experiment. No available data have been excluded from any of the data sets.

### Cell frequency, counts, and expression of cell surface markers by flow cytometry.

Cells were taken from the monocyte-enriched hyperosmotic Percoll layer and Fc blocked with Human TruStain FcX (BioLegend, catalog 422302) and stained with fluorochrome-conjugated antibodies specific for CD14 (Alexa Fluor 488, BioLegend, catalog 325610), CD68 (Biolegend, catalog 333808), CD16 (PerCP-Cy5.5, BioLegend, catalog 302027), CD80 (PE-Cy7, BioLegend, catalog 305218), CD86 (BV421, BioLegend, catalog 302027), CD40 (BV510, BioLegend, catalog 334330), and HLA-DR (APC, BioLegend, 307610). Cells were acquired on a BD FACSCanto II. Unstained cells and FMO controls were used to normalize for background staining and to set gates. Data were analyzed using FlowJo software.

Monocytes were identified as CD14^^+^^CD68^^–^^ cells, and nonclassical monocytes were identified as being CD14^^+^^CD16^^+^^. To estimate the absolute number of monocytes in each blood preparation, the cell total cell yield from the hyperosmotic Percoll gradient was multiplied by the percentage of CD14^^+^^ cells present in the gradient yield. The frequency of monocytes in the blood was then calculated by dividing the absolute number of monocytes by the total PBMC count. Similarly, to estimate the absolute number of nonclassical monocytes in each blood preparation, the cell total cell yield from the hyperosmotic Percoll gradient was multiplied by the percentage of CD14^^+^^CD16^^+^^ cells present in the gradient yield. The frequency of nonclassical monocytes in the blood was then calculated by dividing the absolute number of nonclassical monocytes by the total PBMC count. The expression of CD14, HLA-DR, CD40, CD80, and CD86 was determined using the MFI of the marker as a surrogate for protein expression. The background MFI of each marker on unstained cells was subtracted from each sample to normalize for differences in the lasers that can occur over time.

### Determination of mRNA transcript levels.

RNA extractions from monocytes were performed using an RNeasy Mini Kit (Qiagen, catalog 74136) according to the manufacturer’s instructions. RNA content and quality were quantified and assayed, respectively, using a Nanodrop (Thermo Fisher Scientific) and RNA reverse transcribed using the SensiFast Reverse-Transcription Kit (Meridian Biosciences, catalog BIO-65054). Cataloged TaqMan (Thermo Fisher Scientific) predesigned gene primer probes attached to the FAM dye were used: *GPI* (Hs00976715_m1), *PFKFB3* (Hs00998698_m1), *GAPDH* (Hs02786624_g1), *PKM2* (Hs00761782_s1), and *ATP5B* (Hs00969569_m1). *18S* (Hs03003631_g1) was used as the reference gene primer, attached to the VIC dye. RT-qPCR was performed using SensiFast Probe Mix (Meridian Biosciences, catalog BIO-82005) on a QuantStudio 5 RT-qPCR System (Applied Biosystems). Relative quantitative data were obtained and analyzed utilizing the 2^^–ΔΔCt^^ method.

### Monocyte stimulation assay.

1 × 10^^5^^ cells were added to flat-bottom 96-well plates (Thermo Scientific, catalog 167008) and allowed to adhere for 1 hour at 37°C. Cells were then washed with warm PBS to wash away nonadherent cells and resuspended in RPMI supplemented with 10% male AB Human Serum (Sigma-Aldrich, catalog H3667) and incubated overnight. The next day, cells were stimulated with LPS (Sigma-Aldrich, catalog L2630; 10 ng/mL) or iH37Rv (gifted by BEI Resources; 10 μg/mL), or Pam3Csk4 (Invivogen, catalog tlr-pms; 10 μg/mL) for 24 hours. Unstimulated cells were assayed in parallel as a control. Supernatants were collected and cryopreserved for analysis by multiplex ELISA.

### Cytokine production by monocytes.

Cytokine and chemokine production by monocytes at 24 hours after stimulation were measured using a multiplex ELISA (Assay Genie, custom plate design) according to the manufacturer’s instructions. Beads were collected on an Amnis CellStream using a plate reader. The cytokines measured included IL-1β, IL-6, TNF, IL-10, GM-CSF, IFN-γ, IL-18, IL-4, IL-8, IL-12p70, IL-23, and IFNα2. The chemokines measured included MCP-1, CXCL1, CXCL2, and MIP-1α.

### Statistics.

Data were analyzed using GraphPad Prism software (version 9). One-way repeated measures ANOVA was used to statistically analyze differences in the frequency and absolute number of CD14^^+^^ cells, the frequency of CD14^^+^^CD16^^+^^ cells, and in the MFI of CD14, HLA-DR, CD40, CD80, and CD86 following vaccination with the ChAdOx1 nCoV-19 vaccine. Statistically significant differences between the expression of mRNA transcripts of *GPI*, *PFKFB3*, *GAPDH*, *PKM2*, and *ATP5B* were determined by a mixed-effects model (restricted maximum likelihood [REML]) ANOVA with Šídák’s multiple comparisons test. For the production of cytokines and chemokines using multiplex ELISA, statistically significant differences were assessed using repeated measures 1-way ANOVA with appropriate post tests. *P* values of less than 0.05 were considered significant.

### Study approval.

This study was approved by the Faculty of Health Science Research Ethics level 2 Committee, Trinity College Dublin. Written informed consent was received prior to participation, in accordance with the EU’s General Data Protection Regulation and Health Research Regulations.

## Author contributions

Authorship was assigned based on International Committee of Medical Journal Editors criteria. DMM provided conceptualization, methodology, formal analysis, investigation, data curation, visualization, and project administration and wrote the original draft of the manuscript and reviewed and edited it. DJC provided conceptualization, methodology, and investigation and reviewed and edited the manuscript. SAC provided formal analysis, investigation, and data curation and reviewed and edited the manuscript. EPB provided methodology, resources, formal analysis, and investigation and reviewed and edited the manuscript. AAIB provided investigation and reviewed and edited the manuscript. JJP provided investigation and reviewed and edited the manuscript. JK provided methodology and resources, reviewed and edited the manuscript, and acquired funding. SAB provided conceptualization, methodology, data curation, visualization, supervision, and project administration; wrote the original draft of the manuscript and reviewed and edited it; and acquired funding. All authors have approved the final version of the manuscript to be published and agreed to be accountable for all aspects of the work.

## Supplementary Material

Supplemental data

Trial reporting checklists

ICMJE disclosure forms

## Version 1 10/25/2022

In-Press Preview

## Version 2 01/17/2023

Electronic publication
