# Supplementary material for: Trained immunity is induced in humans after immunization with an adenoviral vector COVID-19 vaccine
Source: J Clin Invest. 2023 Jan 17;133(2):e162581. doi: 10.1172/JCI162581 (PMC9843058; doi:10.1172/JCI162581)
Supplement: Supplemental data [file jci-133-162581-s031.pdf]

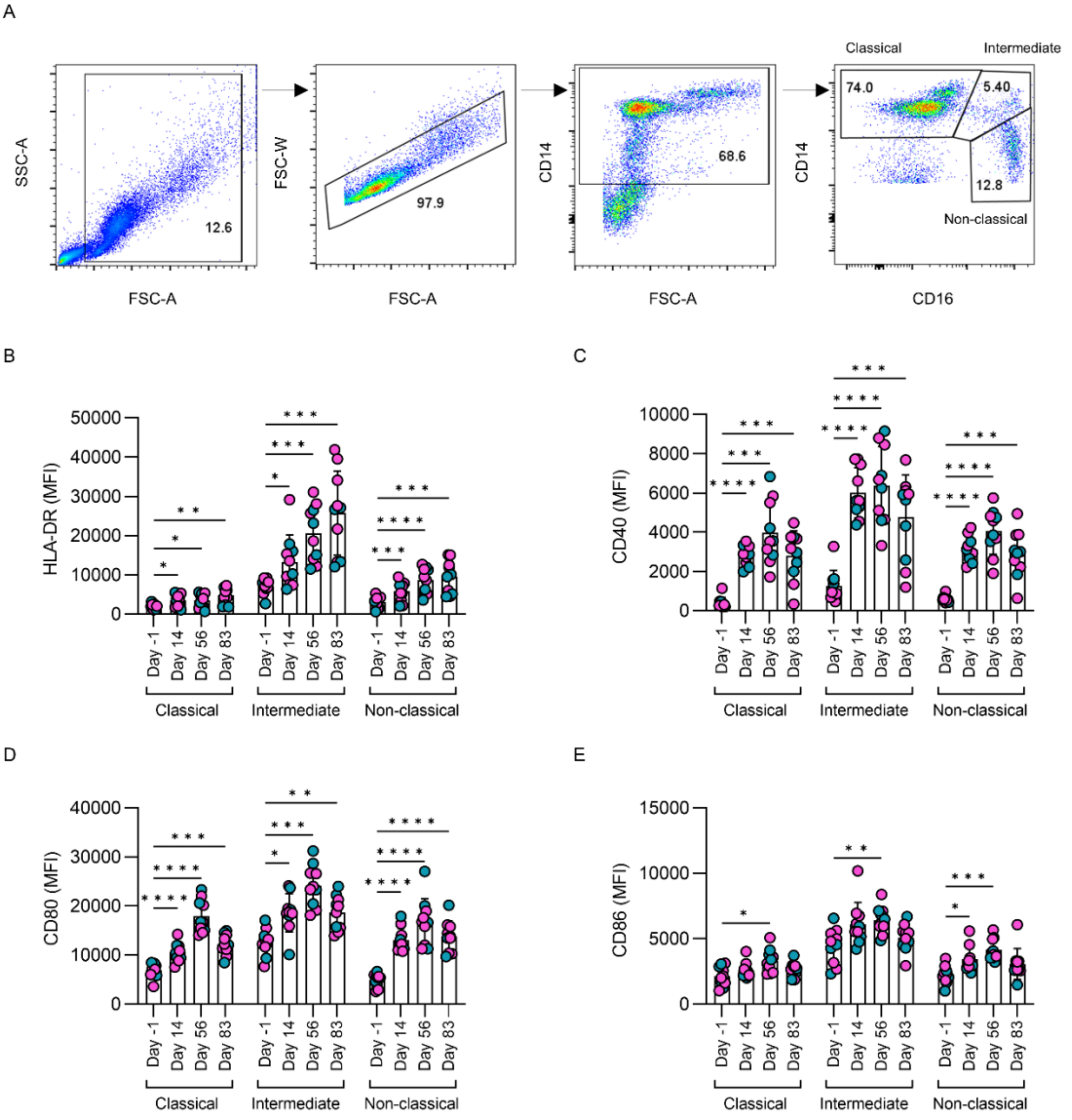

Supplemental Figure 1: Expression of HLA-DR and co-stimulatory molecules on classical, intermediate and non-classical monocytes. Monocytes were isolated from the PBMC of healthy donors on day -1 (pre-vaccine), day 14, day 56 and day 83 after vaccination using a hyperosmotic percoll gradient. Cells were Fc blocked and stained with fluorochrome-conjugated antibodies specific for CD14, and CD16. Classical monocytes were identified as $CD14^{+}$ $CD16^{-}$, intermediate monocytes were identified as $CD14^{+}$ $CD16^{+}$, and non-classical monocytes were identified as $CD14^{lo}$ $CD16^{+}$ with the gating strategy shown (**A**). The cell surface expression of the antigen presentation marker HLA-DR (**B**) and the T cell co-stimulatory molecules CD40 (**C**), CD80 (**D**) and CD86 (**E**) on all three monocyte subsets was assessed ex vivo by flow cytometry. Each dot represents an individual donor (n=10) with blue dots denoting male donors and pink dots denoting female donors. Data is graphed as the mean value ± SD. Statistically significant differences between the groups were determined by a repeated measures two-way ANOVA using Dunnett's multiple comparisons test; ****P<0.0001, ***P<0.001, **P<0.01, *P<0.05.

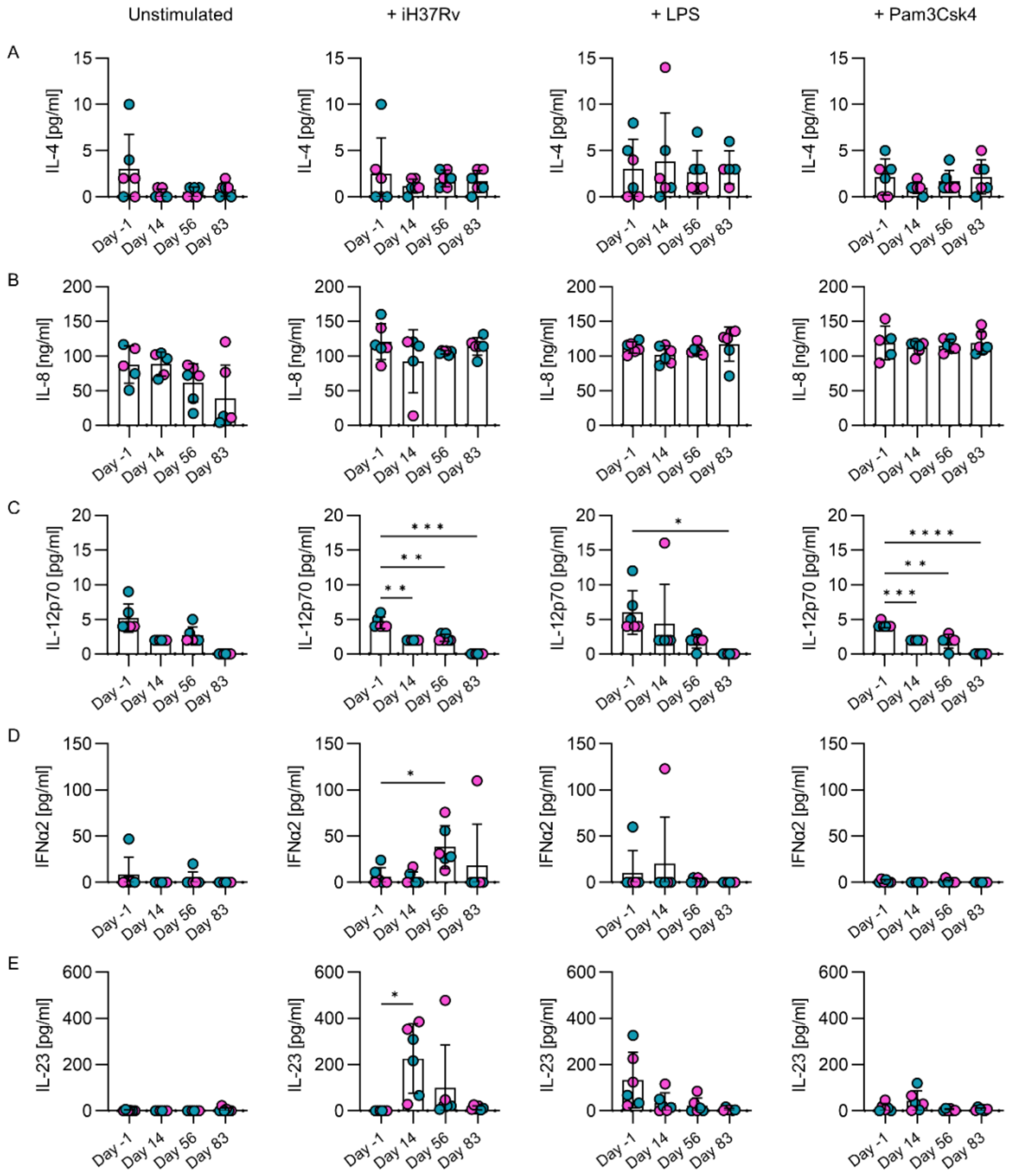

Supplemental Figure 2: Cytokine production from monocytes pre and post vaccination. Monocytes were enriched from the PBMC of healthy donors on the day before (day -1) and day 14, 56 and 83 after vaccination using a hyperosmotic percoll gradient. Monocytes were further purified using plastic adherence and were routinely over 90% pure. Monocytes were left to rest overnight and stimulated ex vivo with medium (unstimulated), irradiated *M. tuberculosis* (iH37Rv; 10 µg/ml), LPS (10 ng/ml), or Pam3Csk4 (10 µg/ml) for 24 hours. The concentrations of IL-4 (**A**), IL-8 (**B**), IL-12p70 (**C**), IL-23 (**D**), and IFNα2 (**E**) was assessed using multiplex ELISA on cell supernatants with (**A, C-E**) showing [pg/ml] and (**B**) showing [ng/ml]. Each dot represents an individual donor (n=6) with blue dots denoting male donors and pink dots denoting female donors. Data is graphed as the mean value ± SD. Statistically significant differences between the groups were determined by a repeated measures one-way ANOVA using Dunnett's multiple comparisons test; ****P<0.0001, ***P<0.001, **P<0.01, *P<0.05.
